# Management of Mycotic Abdominal Aortic Aneurysm Caused by Levofloxacin- and Ampicillin-Resistant Campylobacter fetus With Synthetic Vascular Graft Replacement Surgery and Long-Term Minocycline Therapy

**DOI:** 10.7759/cureus.83533

**Published:** 2025-05-05

**Authors:** Jun Makino, Fukumi U Nakamura, Manabu Shiraishi, Kazuya Okada, Takao Goto

**Affiliations:** 1 Department of Critical Care Medicine, Tokyo Metropolitan Bokutoh Hospital, Sumida, JPN; 2 Department of Infectious Diseases, Tokyo Metropolitan Bokutoh Hospital, Sumida, JPN; 3 Department of Cardiovascular Surgery, Tokyo Metropolitan Bokutoh Hospital, Sumida, JPN

**Keywords:** ampicillin, campylobacter fetus, case report, early surgical intervention, levofloxacin, long-term targeted antimicrobial therapy, minocycline, mycotic aortic aneurysm

## Abstract

Mycotic aortic aneurysms caused by *Campylobacter fetus* (*C. fetus*) are rare, and the optimal regimens and duration of antimicrobial therapy remain unclear. We present a case of a man in his 70s with hypertension and chronic heavy alcohol consumption who presented with an eight-day history of persistent fever. Contrast-enhanced computed tomography of the abdomen and pelvis revealed a mycotic infrarenal aortic aneurysm. Empiric ceftriaxone and vancomycin were switched to minocycline after cultures identified levofloxacin- and ampicillin-resistant, but minocycline-susceptible *C. fetus*. The patient underwent synthetic graft replacement surgery on day 8 and completed 16 weeks of postoperative minocycline, achieving full recovery and showing no recurrence at a three-year follow-up. This case highlights the importance of early surgical intervention combined with long-term, targeted antimicrobial therapy for managing *C. fetus*-related mycotic aneurysms.

## Introduction

*Campylobacter fetus* (*C. fetus*), a Gram-negative, microaerophilic bacterium with three subspecies consisting of *C. fetus fetus*, C. *fetus testudinum*, and *C. fetus venerealis*, is one of over 40 *Campylobacter *species and a rare pathogen that primarily infects elderly or immunocompromised individuals through exposure to livestock feces [1,2]. *C. fetus* infections account for 19-53% of *Campylobacter* bacteremia cases and cause a variety of invasive infections, including meningitis, brain abscess, major vascular infections, infective endocarditis, pulmonary abscess, osteomyelitis, and arthritis [1,2]. However, the optimal antimicrobial selection and treatment duration for *C. fetus* infections remain unclear, and standard breakpoints for antimicrobial susceptibility testing by the Clinical and Laboratory Standards Institute (CLSI) or the European Committee on Antimicrobial Susceptibility Testing (EUCAST) [1,3,4] are not established. Here, we report a case of a mycotic abdominal aortic aneurysm caused by levofloxacin- and ampicillin-resistant *C. fetus* that was successfully treated with early synthetic graft replacement surgery and long-term oral minocycline therapy.

This article was previously presented as a meeting abstract at the 2024 Eastern Japan Regional Annual Scientific Meeting of the Japanese Association for Infectious Diseases on October 18, 2024.

## Case presentation

The patient was a man in his 70s with a history of hypertension and chronic heavy alcohol consumption. He presented with a fever exceeding 39°C that had persisted for eight days. He reported no respiratory, gastrointestinal, or musculoskeletal symptoms. His medical history included hypertension treated with amlodipine 5 mg once daily and olmesartan 20 mg once daily. He had no other significant medical history or known drug allergies. His social history included daily consumption of approximately 86.4 g of pure alcohol (the ideal alcohol intake in general is 20 g or less per day) since the age of 20 and smoking eight cigarettes daily until the age of 50. He worked in sewage management until the age of 60 and reported no recent travel history, pet ownership, contact with animals, or consumption of raw meat or dairy products. Upon arrival, he was alert and oriented, with a body temperature of 39°C, blood pressure of 146/85 mmHg, pulse rate of 114/min, respiratory rate of 24/min, and oxygen saturation measured by pulse oximetry of 97% on room air.

On physical examination, a pulsatile mass was palpated in the abdomen, while no abnormal findings were observed in the examination of the head and neck, respiratory and cardiovascular systems, extremities, or skin. Initial blood tests (Tables 1-2) revealed elevated inflammatory markers, including a white blood cell count of 9,300/µL (normal range: 3,300-8,600/μL) with 93.7% neutrophils (normal range: 37.0-80.0%) and C-reactive protein level of 13.4 mg/dL (normal range: 0-0.14mg/dL). Additionally, the fibrin degradation product level was elevated, at 13.2 µg/mL (normal range: <5.0 μg/mL), indicating fibrinolytic activity.

**Table 1:** Hematologic and coagulation findings on admission WBC: White blood cell count; Hb: Hemoglobin; Hct: Hematocrit; Plt: Platelet count; PT: Prothrombin time; APTT: Activated partial thromboplastin time; Fib: Fibrinogen; FDP: Fibrin degradation products

Parameter	Lab value	Reference range
WBC (/µL)	9,300	3,300-8,600
Neutrophils (%)	93.7	37.0-80.0
Hb (g/dL)	15.8	13.7-16.8
Hct (%)	46.7	40.7-50.1
Plt (x103/µL)	198	158-348
PT (%)	71.4	80.0-120.0
APTT (sec)	32.7	24.0-39.0
Fib (mg/dL)	565	200-400
FDP (µg/mL)	13.2	<5.0

**Table 2:** Biochemistry findings on admission T-bil: Total bilirubin; AST: Aspartate aminotransferase; ALT: Alanine aminotransferase; LDH: Lactate dehydrogenase; γ-GTP: Gamma-glutamyl transpeptidase; CRP: C-reactive protein; Glu: Glucose

Parameter	Lab value	Reference range
T-bil (mg/dL)	2.55	0.40-1.50
AST (IU/L)	63	13-30
ALT (IU/L)	77	10-42
LDH (IU/L)	217	124-222
γ-GTP (IU/L)	192	13-64
CRP (mg/dL)	13.4	0-0.14
Glu (mg/dL)	164	73-109

Biochemical tests revealed mild elevation of liver enzymes, with aspartate and alanine aminotransferase levels of 63 IU/L (normal range: 13-30 IU/L) and 77 IU/L (normal range: 10-42 IU/L), respectively; mild jaundice with a total bilirubin level of 2.55 mg/dL (normal range: 0.40-1.50 mg/dL); and mild hyperglycemia with a serum glucose level of 164 mg/dL (normal range: 73-109mg/dL).

**Figure 1:**
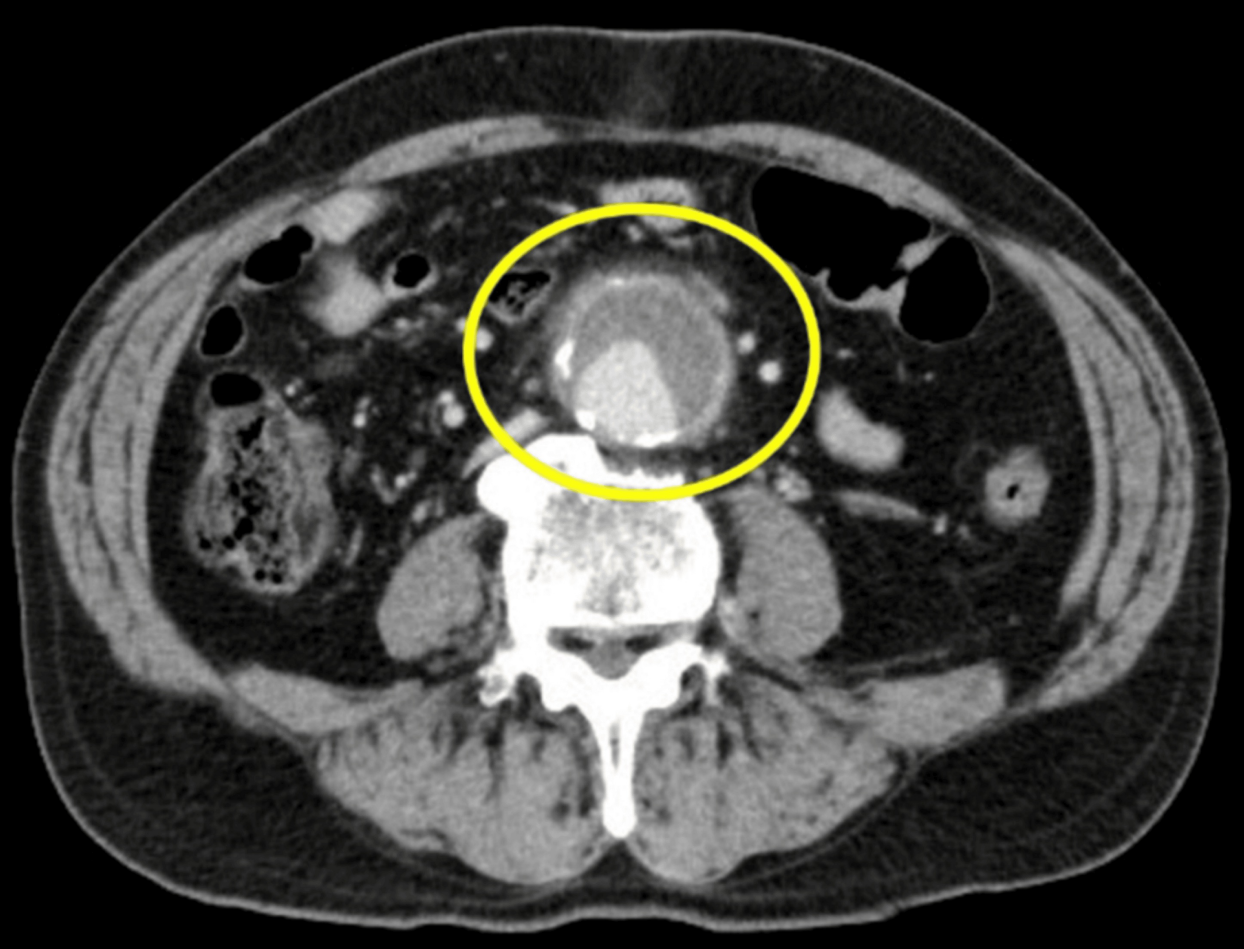
Contrast-enhanced CT of the abdomen and pelvis on admission The scan shows a 48-mm infrarenal abdominal aortic aneurysm (yellow circle) with increased attenuation of the surrounding adipose tissue extending from the anterior to the lateral aspect of the infrarenal aorta.

Urinalysis and plain radiographs of the chest and abdomen showed no significant findings. Contrast-enhanced computed tomography (CT) of the abdomen and pelvis (Figure 1) revealed a 48-mm abdominal aortic aneurysm below the renal arteries with increased adipose tissue density around the lesion, consistent with a mycotic aneurysm.

**Figure 2:**
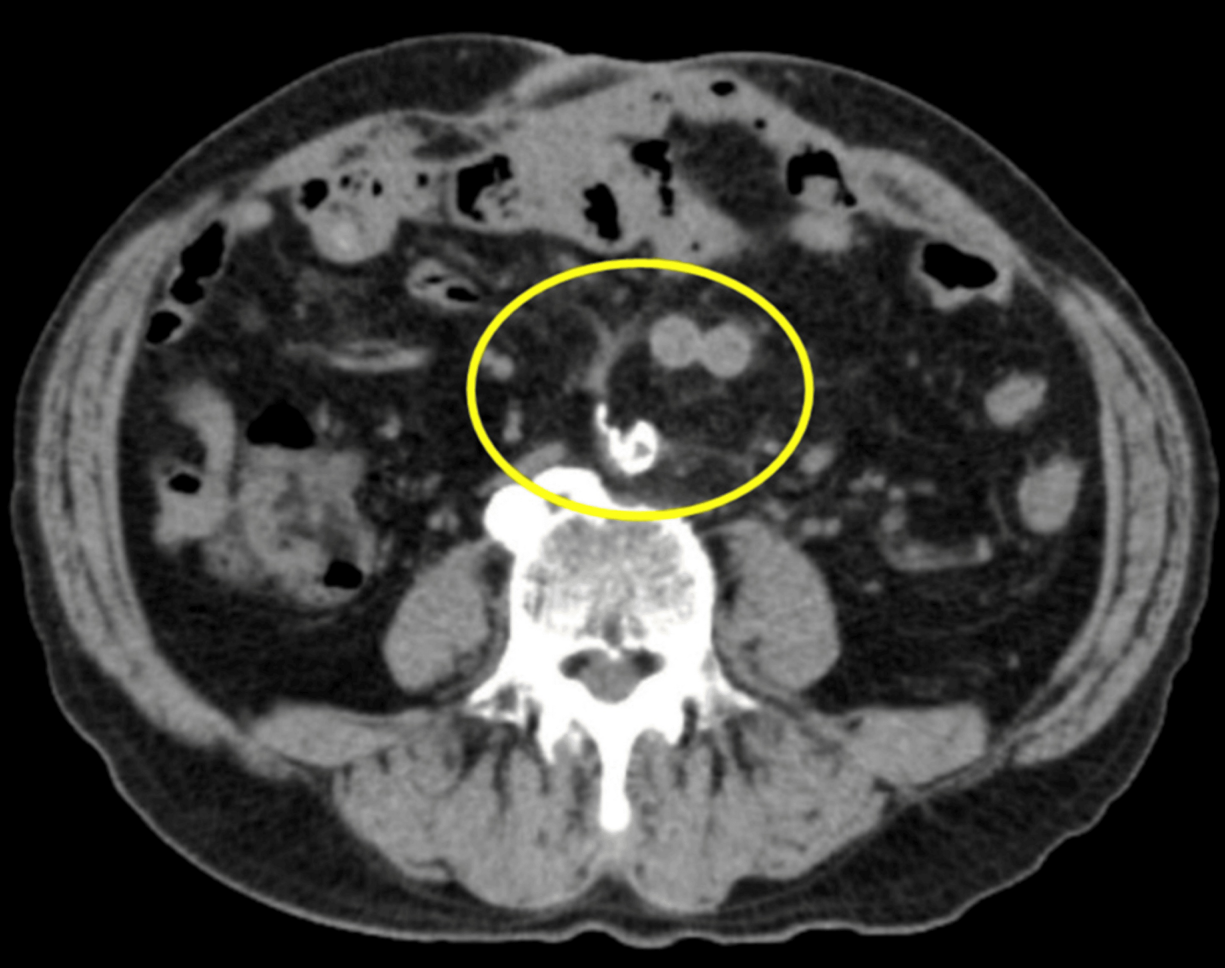
Plain CT of the abdomen and pelvis at 16 weeks The scan shows progressive resolution of fluid accumulation around the prosthetic graft (yellow circle).

Given the risk of a mycotic aneurysmal rupture, the patient was admitted to the intensive care unit for close observation. Blood cultures were obtained on admission, and empiric intravenous ceftriaxone 2 g once daily and intravenous vancomycin were initiated. On day 5, two sets of blood cultures on admission grew spiral-shaped Gram-negative bacilli, which were subsequently identified as *C. fetus fetus *by polymerase chain reaction and matrix-assisted laser desorption ionization-time of flight mass spectrometry (MALDI-TOF MS) (VITEK®2; bioMérieux, Inc., USA) [5,6]. Subsequent Epsilometer tests (E-tests) confirmed susceptibility to erythromycin, minocycline, and gentamicin, and resistance to levofloxacin and ampicillin. Following consultation with the infectious diseases team, the empiric antimicrobial regimen was changed to intravenous minocycline 100 mg twice daily on day 7. On day 8, synthetic graft replacement was performed using a 20 x 10 mm Dacron bifurcated tube graft (Intergard®, Maquet, Rastatt, Germany), along with omental wrapping surgery. Pathological examination of the excised aneurysm showed advanced atherosclerosis and inflammatory cell infiltration into the media. Cultures from the aneurysm wall, thrombus, and surrounding fluid all isolated *C. fetus*, establishing the definitive diagnosis of mycotic abdominal aortic aneurysm caused by *C. fetus*. The patient’s postoperative course was uneventful until discharge on day 30, after which the treatment was switched to oral minocycline 100 mg twice daily. After discharge, the patient attended follow-up appointments at the infectious diseases and cardiovascular surgery outpatient clinics. Eight weeks after initiating antimicrobial therapy, his erythrocyte sedimentation rate normalized. At 16 weeks, his erythrocyte sedimentation rate remained normal, and plain CT of the abdomen and pelvis (Figure 2) showed progressive resolution of fluid accumulation around the prosthetic graft.

Minocycline treatment was completed after a total of 16 weeks of intravenous and oral administration. At one-year follow-up, there were no signs of recurrence, and the patient has remained relapse-free three years after treatment.

## Discussion

This report presents a case of a mycotic abdominal aortic aneurysm caused by *C. fetus* in an elderly man with chronic alcohol use. *C. fetus* can easily cause bacteremia because of its resistance to phagocytosis, mediated by its surface (S) layer protein [7,8]. While the patient’s history did not reveal a definitive infection route, the combination of advanced age, heavy alcohol consumption, and potential immune compromise may have predisposed the patient to gut barrier dysfunction, leading to *C. fetus* bacteremia and mycotic abdominal aortic aneurysm. The cornerstone of mycotic aortic aneurysm treatment is surgical intervention combined with appropriate antimicrobial therapy. Because mycotic aortic aneurysms carry the risk of rapid expansion and rupture, prompt surgical intervention is recommended regardless of the aneurysm size [9]. Surgical treatment options include open surgical resection, endovascular aneurysm repair, and bypass surgery, chosen based on the patient's background and overall condition. In our case, open surgical resection with synthetic graft replacement and omental wrapping was chosen because CT did not reveal a gross abscess around the site of infection, vertebral osteomyelitis, or aorto-enteric fistula, and there was no hemodynamic instability suggestive of rupture.

As of 2025, there is no established standard antimicrobial regimen for *C. fetus*, and antimicrobial breakpoints have not been clearly defined. Some experts advise against the use of azithromycin, the first-line drug for *Campylobacter* infection, for bacteremia or intravascular infections caused by *C. fetus* [10]. In addition, the frequent use of fluoroquinolones in animal feed has led to a worldwide problem of drug resistance in *Campylobacter*, and the use of fluoroquinolones is not recommended until drug susceptibility is determined. In a nationwide study conducted by Zhang et al. from 2012 to 2023 on the antimicrobial susceptibility of *C. fetus*, the organism showed good susceptibility to meropenem and gentamicin, while reduced susceptibility to ciprofloxacin and tetracyclines was observed in some strains [1]. In the present case, the isolate was susceptible to minocycline, erythromycin, and gentamicin but resistant to ampicillin and levofloxacin (drug susceptibility to meropenem was not assessed). Among these options, minocycline was chosen as targeted therapy based on drug susceptibility and the convenience of continuing treatment on an outpatient basis. However, given the severity of the case, the use of gentamicin or meropenem might have been justifiable in retrospect. Although invasive* C. fetus* infections are relatively rare, antimicrobial therapy has a significant impact on patient outcomes. Therefore, the establishment of standardized antimicrobial breakpoints for *C. fetus* is warranted both domestically and internationally.

The optimal duration of antimicrobial therapy for mycotic aneurysms remains uncertain. The 2016 American Heart Association guidelines on mycotic aneurysms [9] recommend at least six weeks to six months of antimicrobial therapy postoperatively. The 2024 European Society for Vascular Surgery guidelines on mycotic aneurysms [11], excluding prosthetic vascular infections, do not recommend a specific duration for antimicrobial therapy; however, they define resolution as the absence of recurrence for one-year, and recommend close monitoring during this period, including clinical assessments, blood tests, and contrast-enhanced CT. This is because 80-90% of relapses occur within one year of the initial onset, with a mortality rate of up to 50% for relapses occurring within six months [12]. In general, the period from the initial diagnosis to relapse or persistent infection in invasive *C. fetus* infections ranges widely from 20 days to seven years [2]. Based on these results, the 16 weeks of antimicrobial therapy followed by the one-year follow-up in the outpatient clinic in our case can be considered adequate to prevent recurrence.

## Conclusions

Mycotic aortic aneurysms caused by *C. fetus* are rare, and the optimal therapeutic strategy is not established. Prompt surgical synthetic graft replacement followed by appropriate antimicrobial therapy should be continued for six weeks to six months, ideally guided by minimum inhibitory concentration testing. Also, long-term follow-up with clinical assessments, blood tests, and imaging is essential, with a minimum monitoring period of one year to ensure no recurrence.
